# Time-Restricted Feeding Affects Energy Metabolism in Lactating Striped Hamsters (*Cricetulus barabensis*, *Cricetidae*, *Rodentia*)

**DOI:** 10.3390/biology14091261

**Published:** 2025-09-12

**Authors:** Wenting Li, Xinyuan Dong, Jiachen He, Xiaojie Jin, Binxin Yin, Tingbei Bo, Jing Wen

**Affiliations:** 1 School of Life and Environmental Sciences, Wenzhou University, Wenzhou 325035, China; 2 Key Laboratory for Water Environment and Marine Biological Resources Protection in Zhejiang Province, Wenzhou 325035, China; 3 School of Grassland Science, Beijing Forestry University, Beijing 100083, China

**Keywords:** food intake, lactation, metabolic rate, gut microbiota, energy demands

## Abstract

Lactation is a critical period in which mothers generally increase food intake and metabolism to meet high energy demands. In the present study, we examined the effect of time-restricted feeding (TRF) on lactating striped hamsters. On the day of litter birth, lactating females were assigned into 3 experimental groups that experienced 24-h ad lib feeding (Con), 12-h daytime feeding (DF), or 12-h nighttime feeding (NF). A group of non-lactating females with 24-h feeding (NL) served as an additional control. Our data show that lactating females had increased food intake, oxygen consumption, and small intestine mass but a decreased level of circulating melatonin, compared to the NL females. However, TRF manipulation significantly reduced the mother’s food intake, the pup’s body mass, and survival rate. In addition, TRF had some phase-specific (i.e., DF vs. NF) effects on facilitating body mass loss, decreasing *CART*, *AgRP*, and *POMC* gene expression in the hypothalamus, and increasing abundance in *Desulfobacterota* and *Actinobacteriota* in the gut microbiota of lactating females. Together, our data illustrate adaptive changes of lactating hamsters under TRF conditions, and highlight the importance of food access and dietary rhythm regulation in maternal and offspring health, development, and reproductive success. These findings not only expand our understanding of lactation biology and ecological feeding strategies but also highlight the significance of regular dietary patterns for lactating individuals, with particular emphasis on shift workers and other populations with irregular daily schedules.

## Simple Summary

To investigate the effects of time-restricted feeding (TRF) on lactating striped hamsters, we compared four experimental groups: lactating females with 24-h ad libitum feeding (Con), lactating females with 12-h daytime feeding (DF), lactating females with 12-h nighttime feeding (NF), and non-lactating females with 24-h ad libitum feeding (NL). TRF produced phase-specific (i.e., DF vs. NF) effects, with the DF group showing greater changes in maternal body mass, food intake, feeding-related neuropeptides, gut microbiota, and pup body mass and survival compared with the NF group. These results enhance our understanding of lactation biology and ecological feeding strategies and highlight the importance of regular dietary patterns for lactating individuals.

## 1. Introduction

Diet, its nutritional quality, and feeding patterns play important roles in body composition, energy balance, and an individual’s health [1,2]. An animal’s feeding pattern is usually associated with its biological activities, with food intake and body mass fluctuating according to the biological rhythms [3,4]. For example, nocturnal mice under nighttime-feeding with a high-fat diet can effectively attenuate their body mass gain, whereas ones with daytime-feeding markedly elevate their body mass and perturb circadian metabolic rhythms [5]. Data have also shown that skeletal muscle mitochondrial respiration exhibits significant diurnal rhythmicity in nighttime-fed rats, and conversely, for daytime-fed rats, the mitochondrial rhythmicity is disrupted [6]. In general, when feeding rhythms align with the animal’s biological rhythms, such alignment can enhance circadian clock synchronization, improve mass regulation, and promote metabolic homeostasis—a phenomenon which has been observed in rats (*Rattus norvegicus*, Berkenhout, 1769), greater mole-rats (*Spalax microphthalmus*, Güldenstädt, 1770), and mice (*Mus musculus*, Linnaeus, 1758) [7,8,9,10]. However, time-restricted feeding (TRF) seems to have no effects on body mass and metabolic rate in Mongolian gerbils (*Meriones unguiculatus*, Milne-Edwards, 1867) [11], suggesting that species differences do exist and thus data from one species should not be simply extrapolated to another.

TRF is a commonly used experimental manipulation of feeding that limits the daily feeding period (usually to ≤12 h/day) within a certain time during the circadian rhythm. TRF has been shown to impose no explicit limitations on the quality and quantity of food intake [12,13] and can even improve energy metabolism, increase gut microbiota richness, and strengthen circadian rhythm [14]. TRF has also been shown to alter a variety of biological markers from their normal rhythmic patterns. For example, melatonin exhibits circadian rhythmicity, which is lower in the daytime and higher at night [15]. Melatonin serum levels were 3.5-fold higher in the late eating population versus the early condition [16]. Cryptochrome 1 (Cry1), Brain and Muscle ARNT-Like 1 (Bmal1), and Nuclear Receptor Subfamily 1, Group D, Member 1 (NR1D1) proteins play vital roles in regulating circadian rhythms and metabolism [17,18,19,20]. These gene expressions exhibit distinct circadian rhythms, with Cry1 and NR1D1 showing higher levels in the day and lower levels at night, while Bmal1 displays the opposite pattern. TRF improves hepatic circadian rhythms by regulating the expression of clock genes such as *Bmal1*, *Rev-erbα/β*, and *Clock* [21,22,23]. Among them, *Bmal1* is synchronized with feeding times and may influence feeding behavior by regulating energy metabolism and appetite in mice [24,25], whereas daytime-restricted feeding reduced Cry1 expression in the hypothalamus and liver for Mongolian gerbils (*Meriones unguiculatus*) [11]. Additionally, the gut microbes also exhibited clear circadian oscillations, which synchronized with the host’s circadian rhythm [26]. TRF manipulation alters gut microbiota rhythmicity, which in turn affects host metabolism [27,28]. These findings suggest that dietary patterns are closely linked to the rhythmicity of the gut microbiota. Daytime feeding led to muscle loss, impaired gut barrier function, and induced dysbiosis of gut microbiota in mice [29]. Specifically, it disrupted the rhythms of *Akkermansia*, *Lactobacillus*, and *Bacteroides* in C57BL/6 mice [30]. However, previous research has primarily focused on the effects of TRF on adult individuals and has rarely paid attention to the specific life history stages of animals.

Lactation is a critical period of high energy demand, and lactating mothers are thus more vulnerable and susceptible to negative environmental impacts [31,32]. For example, lack of nutritional food or limited food intake is related to the disruptions of the activities, physiological markers, and milk production of the mothers, ultimately affecting their reproductive success [33,34]. In addition, studies have shown that lactation also significantly affects circadian rhythms in mice—the amplitude and robustness of the expression of circadian genes in lactating C57BL/6J mice are significantly enhanced in the liver (*Per2*, *Cry1*, and *Cry2*) [35]. Interestingly, peripheral clocks can also be entrained indirectly by feeding rhythms [36], and TRF manipulations can interfere with the rhythm of the body’s activity and physiological indicators [37,38,39]. However, the effects of TRF on maternal energy metabolism during lactation, through disrupted feeding rhythms, remain unclear.

The striped hamster (*Cricetulus barabensis*, Pallas, 1773) is primarily found in the farmlands and grasslands of northern and northeastern China [40]. As nocturnal animals, they exhibit clear circadian rhythmic activities in their locomotion, feeding, and other behavioral patterns as well as a variety of physiological and metabolic markers [41]. Data have also shown that under a 12L:12D photoperiod, the circadian oscillations in the expression of clock genes, melatonin receptor genes, and metabolic genes in central and peripheral tissues of female striped hamsters are involved in regulating their biological rhythms and metabolic homeostasis [42]. Although previous studies examined the effects of environmental stress and food quality on the energy metabolism of lactating striped hamsters [31,43], little attention has been paid to the impacts of TRF during lactation. Therefore, the present study aims to use the female striped hamster as a model to investigate the effects of TRF during lactation on maternal metabolic homeostasis. In addition to a variety of physical, metabolic, and behavioral measurements, we also assessed the expression of several neuropeptide genes, serum melatonin, gut microbiome, and circadian clock proteins to have a better understanding of the bodily impacts of the disrupted feeding patterns on lactation-stripped hamsters. We hypothesized that TRF would disrupt the adaptive energy allocation of lactating striped hamsters, thereby further impairing maternal performance and offspring development.

## 2. Materials and Methods

### 2.1. Experimental Animals

Female striped hamsters (around 2 months of age) were housed in plastic cages (29 cm × 18 cm × 16 cm) with sawdust bedding. The room temperature was maintained at 23 ± 1 °C, the light-dark cycle was set to 12L:12D (lights on at 8:00 a.m.), and water and food (containing 4.0% crude fat, 18.0% crude protein, 5.0% crude fiber, and an energy value of 17.6 kJ/g, provided by Beijing Keao Xieli Feed Co., Ltd., Beijing, China) were supplied ad libitum. Females were paired with age-matched males to get pregnant, and once pregnancy was confirmed, the males were removed, leaving the females as experimental subjects. Ultimately, the females that were unable to conceive were also separated from the males. The animal care and experimental procedures were approved by the Animal Care and Use Committee of Wenzhou University (WZU-2022-085).

### 2.2. Experimental Design

**Figure 1:**
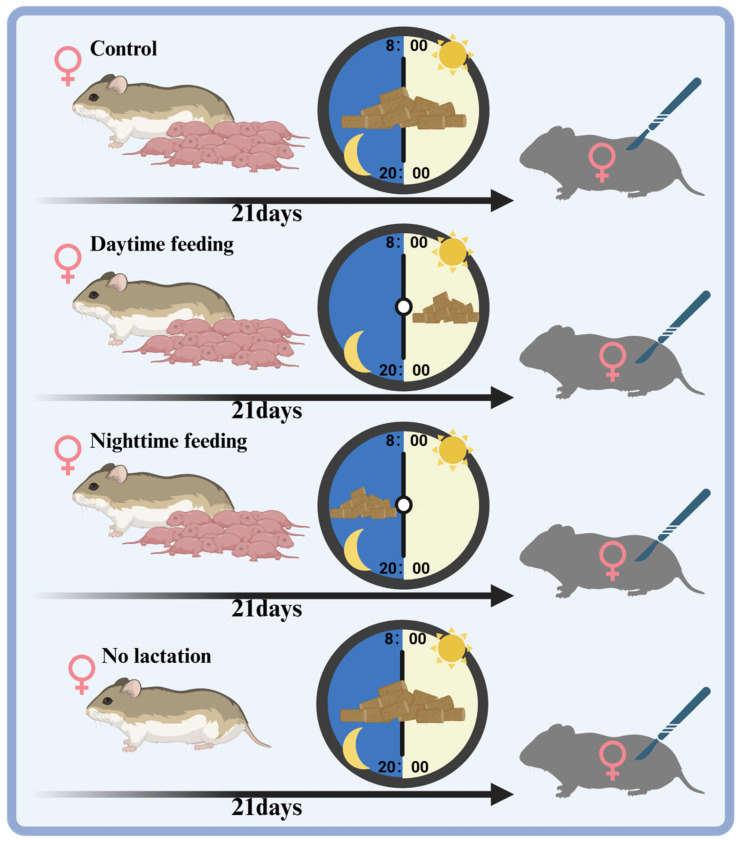
Schematic illustration for time-restricted feeding acclimatization in female striped hamsters.

Pregnant striped hamsters were individually housed once pregnancy was visually confirmed via abdominal distension, and this individual housing was maintained until parturition, after which, dams were cohoused with their offspring until weaning. From the first day of litter birth (without human intervention), mothers and their pups were randomly assigned into one of the following feeding conditions: ad libitum feeding (Con, 24 h feeding, n = 8), daytime feeding (DF, 8:00–20:00 feeding, n = 8), or nighttime feeding (NF, 20:00–8:00 feeding, n = 8) under a 12L:12D photoperiod (lights on at 8:00). These conditions were maintained from the first day after litter birth until the pups were weaned on day 21, at which point they naturally transitioned to solid food. An additional group of non-pregnant (non-lactating, NL) female hamsters that were also individually housed for 21 days under the ad libitum feeding condition (NL, 24 h feeding, n = 8) was also generated as a negative control (Figure 1).

### 2.3. Measurement of Mother Body Mass, Litter Body Mass, and Dietary Intake

The body mass was measured using a balance (Sartorius, Germany) with an accuracy of 0.1 g. At the start of the experiment (postnatal day 1), the number of litters was recorded. The maternal and litter body mass and food consumption (to within 0.1 g) were measured daily at 8:00 and 20:00 for 20 days. Daily food consumption was calculated as the difference between the total mass of food provided and the food remaining in the feeder, minus any food debris scattered in the bedding (spilled food was separated from bedding through manual observation and then weighed). Care was taken to minimize any potential disturbance to the animals during handling.

### 2.4. Measurement of Metabolic Rate

**Figure 2:**
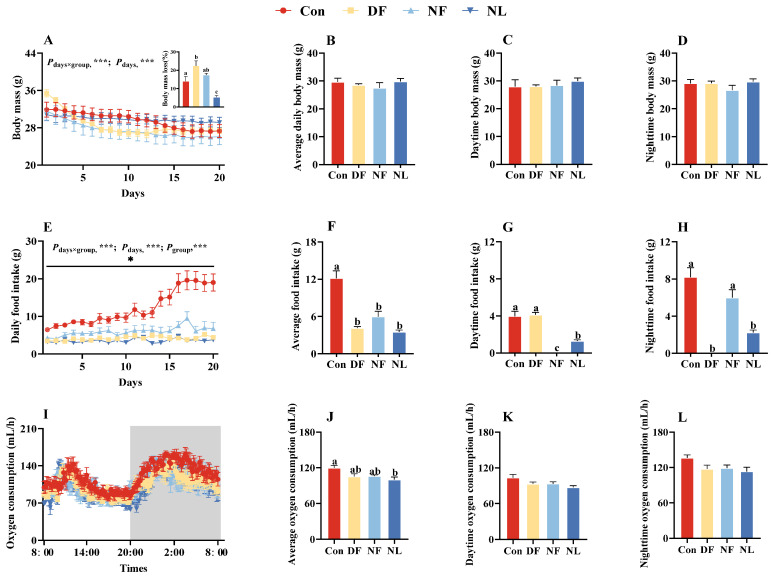
Effects of time-restricted feeding on body mass, food intake, and metabolic rate in female striped hamsters. Body mass showed a continuing decline during the 21-day period, and the DF group showed a higher body mass loss (the bar graph at the top right corner of (**A**) than Con group (**A**), but no group differences were found on daily average body mass (**B**), daytime body mass (**C**), and nighttime body mass (**D**). Food intake changed during the 21-day period in a group-dependent manner (**E**). Group differences were also found on daily food intake (**F**), daytime food intake (**G**), and nighttime food intake (**H**). Metabolic rates fluctuated during the 24-h measurement (**I**). Con group showed a higher average metabolic rate than the NL group (**J**), whereas no group differences were found on daytime metabolic rate (**K**) and nighttime metabolic rate (**L**). Con: control group; DF: daytime feeding group; NF: nighttime feeding group; NL: non-lactating group. Data are presented as means ± standard error. * *p* < 0.05, *** *p* < 0.001. Different letters indicate significant intergroup differences determined by Tukey’s post hoc test.

On postnatal day 21, the pups were weaned, and striped hamster mothers were placed individually in a uniform lighting environment with free access to food and water to measure their 24-h metabolic rate (Figure 2I). The daily metabolic rate (DMR) of the lactating hamsters was measured using an open-flow oxygen respirometer (TSE, Baden-Württemberg, Germany). Air was supplied to the temperature-controlled chamber at a flow rate of 1000 mL/min, and dried gas was sampled and analyzed at a flow rate of 380 mL/min using an oxygen analyzer. Metabolic rate was quantified based on oxygen consumption, and data were analyzed using standard analysis software connected to the system (LabMaster Mouse, TSE, Baden-Württemberg, Germany). The DMR measurement temperature was set to 23.0 ± 1 °C (consistent with the acclimation temperature of the striped hamsters), and the measurements were conducted for 24 h, and DMR was expressed as oxygen consumption rates, corrected to standard atmospheric pressure.

### 2.5. Body Tissues and Organs

Surgical scissors, forceps, and an operating table were sterilized by wiping with 75% ethanol to prevent cross-contamination. Additionally, sterile, enzyme-free centrifuge tubes were prepared for storing the excised tissues. At 13:00 on day 23, one day after the end of the metabolic assay, the animals were sacrificed by dislocating the cervical vertebrae, after which their blood and cecal contents were collected. In addition, the animals’ interscapular brown adipose tissue (BAT), subcutaneous fat, brain, muscle, small intestine, liver, carcass (accurate to 0.001 g), and cecum contents were isolated and weighed. The samples were then immediately frozen in liquid nitrogen and subsequently transferred to a −80 °C freezer for subsequent analysis.

### 2.6. Melatonin Measurement

At 13:00 on day 23, one day after the ending of the metabolic assay, blood was collected immediately after the hamster was sacrificed by dislocating the cervical vertebrae. The blood samples were left undisturbed for 3 h at 4 °C. The samples were then centrifuged at 3500 rpm for 10 min to separate the serum, which was stored in a −20 °C freezer for later analysis. Serum melatonin concentrations were measured using a commercially available Mouse MT ELISA Kit (Jingmei Biotechnology, Yancheng, China).

### 2.7. Measurement of Carcass Fat Content

The carcasses of the striped hamster were placed in an oven at 60 °C and dried for 1 week. Once completely dried, the carcasses were weighed using an electronic balance (accurate to 0.001 g) to obtain the dry carcass mass, and approximately 1.00 g of the fully ground sample was placed into a filter paper bag. The weights of the filter paper (pre-dried in a 60 °C oven) and the sample (accurate to 0.001 g) were recorded separately. The filter paper bag containing the sample was placed into a Soxhlet extractor filled with petroleum ether for fat extraction. Once the petroleum ether became clear after refluxing, the filter paper bag was removed and placed in a 60 °C oven to dry for 24 h. It was then weighed using an electronic balance (accurate to 0.001 g). The carcass fat content was calculated using the following formula [44]:

Carcass fat content (%) = (filter paper mass + sample mass − mass of filter paper bag after extraction)/sample mass × 100%

### 2.8. Real-Time PCR

Tissue total RNA was extracted with TRIzol, its quality was determined, and the concentration of the extracted RNA was measured. The RNA from the tissues was used as a template, and cDNA was synthesized by reverse transcription using a reverse transcription reagent; 50 μL of cDNA was synthesized by reverse transcription using 2 μL of RNA as a template, and (dT)18 was used as a random reagent. The reaction system was as follows: 2 μL cDNA template, 10^−12^ L SYBR Premix EX Tag TM (2×), 0.4 μL each of upstream and downstream primers (Table 1), and the remainder of the reaction was made with DEPC water to give a total of 20 μL of the system. System setup was: 95 °C predenaturation for 30 s, PCR reaction: 95 °C denaturation for 5 s, 55 °C annealing for 30 s, 72 °C denaturation for 30 s, and the reaction is 40 cycles.

### 2.9. Oroboros O2K Analysis of Mitochondrial Energy Metabolism in Tissues

A total of 50 mg of liver tissue was cut up and mixed with 500 μL of MIR05 respiratory medium with the use of a manual glass homogenizer. The homogenate was homogenised up and down 6 times to obtain a homogenate. The stopper of the sample chamber was removed, 100 μL of tissue homogenate was added to the sample chamber of the Oroboros O2K Cellular Energy Metabolism Assay System by pipetting, the MIR05 respiratory culture was added to replenish to a total volume of 2 mL, and the chamber was closed for assay. The instrument operated at 37 °C with an oxygen flow resolution of 1 pmol O_2_∙s^−1^∙mL^−1^. During the measurement, a homogeneous sample solution and the baseline of hepatic oxygen consumption in the absence of any reagents were ensured and recorded. The titration sequence is as follows: pyruvate, malate, and glutamate(G+M+P) → ADP(D) and MgCl_2_ → cytochrome c(C) → succinate(S) → UCCP → rotenone(ROT) → antimycin a(Ama) → trimethylpentanediol and ascorbic acid(TMPD+AS). Measured parameters include complex I leak respiration, OXPHOS capacity of Complex I, OXPHOS capacity of Complex I and II, electron transfer system capacity of Complex I and II, the electron transfer system capacity of Complex II, residual oxygen consumption, and OXPHOS capacity of Complex IV. Finally, analysis of changes in oxygen consumption rate in the liver tissue after the addition of different reagents was performed to assess mitochondrial respiratory function.

### 2.10. 16S rDNA Gene Sequencing Analysis

Total DNA was extracted from the cecal content samples using a CTAB-based method combined with phenol–chloroform–isoamyl alcohol extraction and spin-column purification. The V4 region of the bacterial 16S rRNA gene was amplified using specific primers (515F-806R) with barcode sequences through PCR reactions containing Phusion^®^ High-Fidelity PCR Master Mix, primers, and approximately 10 ng of template DNA. Thermal cycling was performed with an initial denaturation at 98 °C for 1 min, followed by 30 cycles of denaturation at 98 °C for 10 s, annealing at 50 °C for 30 s, and extension at 72 °C for 30 s, ending with a final extension at 72 °C for 5 min. PCR products were purified by magnetic bead purification, quantified, pooled in equimolar concentrations, and subjected to Illumina high-throughput sequencing. Paired-end reads were demultiplexed, and primer sequences were trimmed before being merged using FLASH 1.2.11 software. Quality filtering was performed with fastp to generate high-quality clean tags, and chimeric sequences were removed using vsearch based on the SILVA 138.1 or UNITE databases. Denoising was carried out using the DADA2 algorithm implemented in QIIME2 (version QIIME2-202202) to obtain Amplicon Sequence Variants (ASVs). Taxonomic assignment was performed against the SILVA 138.1 database for 16S/18S sequences and the UNITE database for ITS sequences.

### 2.11. Western Blotting

Western blots of whole tissue lysates were probed with primary antibodies: Cry1 (13474-1-AP, Proteintech Group, Wuhan, China), Bmal1 (14268-1-AP, Proteintech Group, Wuhan, China), anti-NR1D1 (14506-1-AP, Proteintech Group, Wuhan, China), and GAPDH (10494-1-AP, Proteintech Group, Wuhan, China). The second antibody was goat anti-rabbit IgG (D111018, Sangon Biotech, Shanghai, China). The procedures were consistent with those described in the prior publication [45]. Relative protein expression levels were determined by normalizing the gray value of the target protein to that of the internal reference protein for statistical analysis (fluorescent and chemiluminescence gel imaging system, Peiqing Science and Technology Co., Ltd., Shanghai, China).

### 2.12. Statistical Analysis

Data were processed and analyzed using SPSS 27.0 software, and results are expressed as mean ± standard error of the mean (mean ± SEM). One-way analysis of variance (ANOVA) was used to compare mean body mass, metabolic rate, tissue mass, melatonin, the expression of hypothalamic feeding—related neuropeptides and the expression of liver circadian proteins among groups under different feeding rhythm conditions. Microbial alpha-diversity indices (Shannon) and taxon abundance matrices were also evaluated through one-way ANOVA. Beta-diversity patterns were visualized through principal coordinates analysis (PCoA) employing both weighted and unweighted UniFrac metrics, alongside ANOSIM permutation testing using the vegan package (version 4.0.3) in an R environment. Differences in daily body mass, litter size, litter mass, and food intake among groups under different food rhythm conditions were analyzed using one-way repeated measures ANOVA. Pearson correlation analysis was used to assess the correlations between hypothalamic neuropeptide mRNA levels and food intake, between clock gene protein expression and food intake, and between metabolic rate and plasma melatonin concentration. All statistical significance was set at *p* < 0.05, and highly significant differences were indicated by *p* < 0.01. One animal died in each of the DF and NF groups, and, therefore, their data were excluded from the data analysis.

## 3. Results

### 3.1. Body Mass, Food Intake, and Metabolic Rate in Lactating Striped Hamster

There were significant interactions between lactation and feeding treatment on maternal body mass (*F*_(57,494)_ = 9.361, *p* < 0.001, Figure 2A) and daily food intake (*F*_(57,494)_ = 9.382, *p* < 0.001, Figure 2E). As lactation progressed, maternal body mass (*F*_(19,494)_ = 75.557, *p* < 0.001, Figure 2A) and food intake showed significant changes (*F*_(19,494)_ = 16.91, *p* < 0.001, Figure 2E). Although there were no significant differences in body mass between the groups (*F*_(3,26)_ = 0.666, *p* > 0.05, Figure 2A), the differences in food intake were significant (*F*_(3,26)_ = 27.016, *p* < 0.001, Figure 2E). Furthermore, there were no significant differences among groups in daily average body mass (*F*_(3,26)_ = 0.666, *p* > 0.05, Figure 2B), daytime body mass (*F*_(3,26)_ = 0.668, *p* > 0.05, Figure 2C), or nighttime body mass (*F*_(3,26)_ = 1.071, *p* > 0.05, Figure 2D). Compared to the Con group, both the DF and NF groups had significantly lower daily food intakes (*F*_(3,26)_ = 27.016, *p* < 0.001, Figure 2F). Additionally, the daytime food intake in the DF group (*F*_(3,26)_ = 40.245, *p* > 0.05, Figure 2G) and the nighttime food intake in the NF group were not significantly different from that of the Con group (*F*_(3,26)_ = 28.481, *p* > 0.05, Figure 2H).

The daily metabolic rate of each group exhibited a distinct diurnal rhythm, characterized by higher levels at night than during the day (Figure 2I). The average metabolic rate (*F*_(3,23)_ = 4.112, *p* < 0.05, Figure 2J), daytime metabolic rate (*F*_(3,23)_ = 2.952, *p* = 0.054, Figure 2K), and nighttime metabolic rate (*F*_(3,23)_ = 2.749, *p* = 0.066, Figure 2L) showed a decreasing trend in both the DF and NF groups compared to the Con group.

### 3.2. Litter Size and Litter Mass

**Figure 3:**
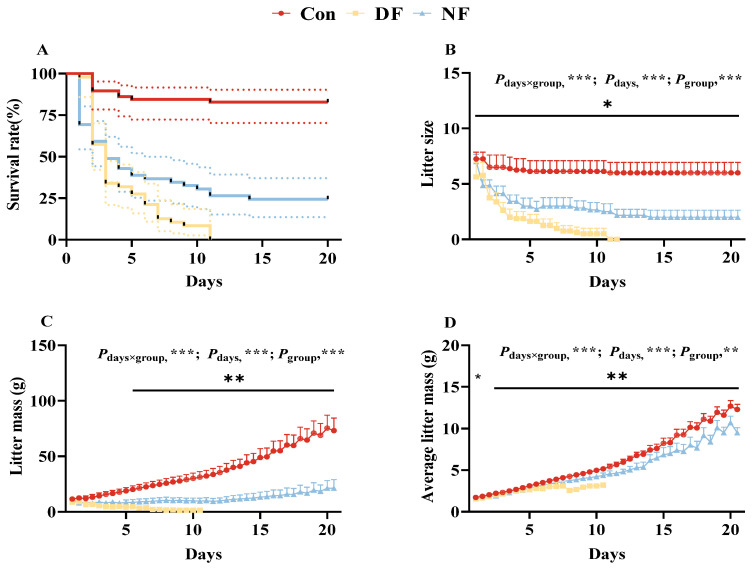
Effects of time-restricted feeding on litter size and litter mass in lactating striped hamsters. Continuous changes in survival rate (**A**), litter size (**B**), total litter mass (**C**), and average litter mass (**D**). Con: control group; DF: daytime feeding group; NF: nighttime feeding group; Data are presented as means ± standard error. * *p* < 0.05, ** *p* < 0.01 and *** *p* < 0.001.

During TRF, maternal infanticide behavior was observed (Figure 3A). Significant interactions between lactation days and feeding treatment were found on the litter size (*F*_(78,741)_ = 6.028, *p* < 0.001, Figure 3B), total litter mass (*F*_(78,585)_ = 45.904, *p* < 0.001, Figure 3C), and average litter mass (*F*_(39,351)_ = 4.733, *p* < 0.05, Figure 3D). With the progression of lactation days, litter size (*F*_(39,741)_ = 35.303, *p* < 0.001, Figure 3B), total litter mass (*F*_(39,585)_ = 51.49, *p* < 0.001, Figure 3C), and average litter mass (*F*_(39,351)_ = 210.454, *p* < 0.001, Figure 3D) showed significant changes. Additionally, both the DF and NF groups had a significantly lower litter size (*F*_(2,19)_ = 14.693, *p* < 0.001, Figure 3B), total litter mass (*F*_(2,15)_ = 52.218, *p* < 0.001, Figure 3C), and average litter mass (*F*_(1,9)_ = 7.762, *p* < 0.05, Figure 3D) compared to the Con group.

### 3.3. Expression of Neuropeptides Involved in Feeding Regulation

**Figure 4:**
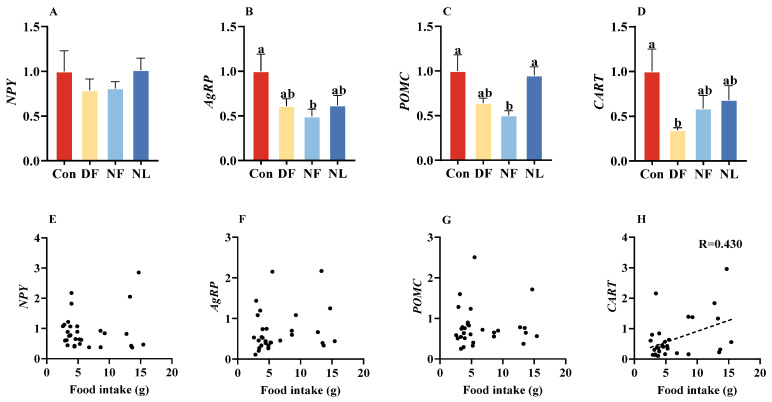
Effects of time-restricted feeding on hypothalamic feeding gene expression in female striped hamsters. No group differences were found in *NPY* mRNA expression in the hypothalamus (**A**), but the Con group showed a higher level of *AgRP* mRNA expression than the NF group (**B**). Con and NL groups also showed higher levels of *POMC* mRNA expression than the NF group (**C**). In addition, the Con group showed a higher level of *CART* mRNA expression than the DF group (**D**). *CART* (**H**), but not *NPY* (**E**), *AgRP* (**F**), and *POMC* (**G**), mRNA expression showed a significant correlation with food intake. Con: control group; DF: daytime feeding group; NF: nighttime feeding group; NL: non-lactating group. Data are presented as means ± standard error. Different letters indicate significant intergroup differences determined by Tukey’s post hoc test.

There is no group difference in the mRNA expression of *NPY* in the hypothalamus of lactating striped hamsters (*F*_(3,56)_ = 0.579, *p* > 0.05, Figure 4A). The NF group exhibited significantly reduced *AgRP* mRNA expression (*F*_(3,56)_ = 2.991, *p* < 0.05, Figure 4B) and *POMC* mRNA expression (*F*_(3,56)_ = 4.327, *p* < 0.01, Figure 4C), whereas the DF group only showed a downward trending. The DF group exhibited a significant reduction in *CART* mRNA expression, whereas the NF group only showed a downward trend (*F*_(3,56)_ = 2.49, *p* = 0.07, Figure 4D). Additionally, a positive correlation was observed between *CART* (*p* < 0.05, Figure 4H) mRNA expression with food intake, while no such correlations were found on *NPY* (*p* > 0.05, Figure 4E), *AgRP* (*p* > 0.05, Figure 4F), and *POMC* (*p* > 0.05, Figure 4G) mRNA expression with food intake.

### 3.4. Organ Weight

**Figure 5:**
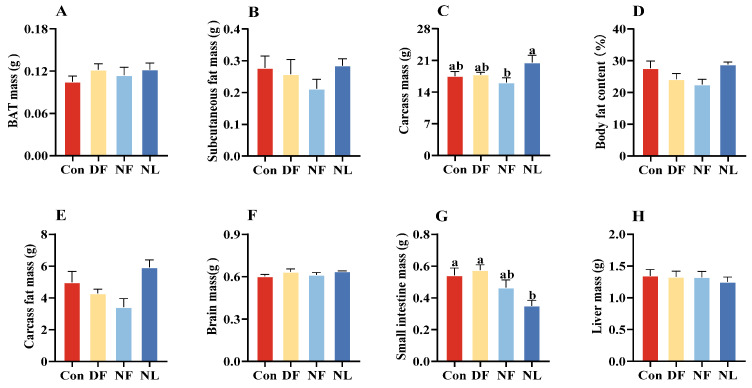
Effects of time-restricted feeding on body composition in female striped hamsters. No group differences were found in the mass of brown adipose tissue (BAT) (**A**), subcutaneous fat (**B**), body fat content (**D**), carcass fat mass (**E**), brain mass (**F**), and liver mass (**H**) in female striped hamster mothers. However, NF females showed lower carcass mass than NL females (**C**). Both Con and DF females showed higher levels of small intestine mass than NL females (**G**). Con: control group; DF: daytime feeding group; NF: nighttime feeding group; NL: non-lactating group. Data are presented as means ± standard error. Different letters indicate significant intergroup differences determined by Tukey’s post hoc test.

There were no significant differences in BAT mass (*F*_(3,26)_ = 0.823, *p* > 0.05, Figure 5A), subcutaneous fat mass (*F*_(3,26)_ = 0.887, *p* > 0.05, Figure 5B), carcass mass (*F*_(3,26)_ = 2.761, *p* = 0.062, Figure 5C), fat content (*F*_(3,26)_ = 2.191, *p* > 0.05, Figure 5D), carcass fat mass (*F*_(3,26)_ = 2.066, *p* > 0.05, Figure 5E), brain mass (*F*_(3,26)_ = 1.344, *p* > 0.05, Figure 5F), and liver mass (*F*_(3,26)_ = 0.247, *p* > 0.05, Figure 5H) across all groups. However, the small intestine mass in lactating striped hamsters was significantly higher than that in the NL group (*F*_(3,26)_ = 6.075, *p* < 0.01, Figure 5G).

### 3.5. Serum Melatonin Levels

**Figure 6:**
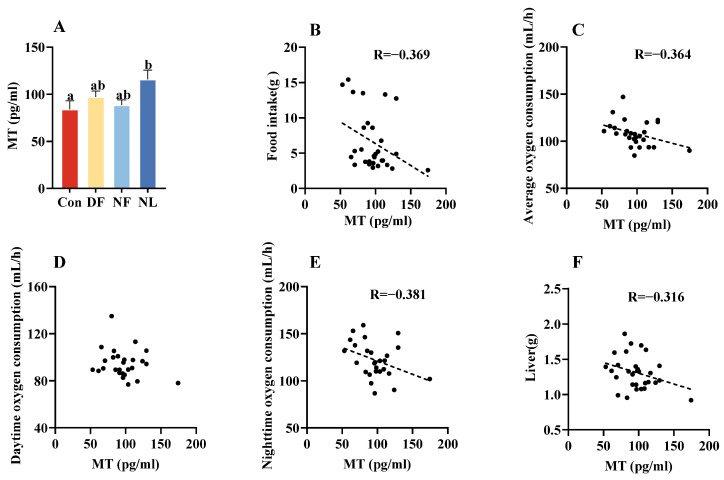
Effects of time-restricted feeding on serum melatonin levels in female striped hamsters. Con females showed a lower level of serum melatonin than NL females (**A**). Food intake (**B**), average metabolic rate (**C**), daytime metabolic rate (**D**), nighttime metabolic rate (**E**), and liver mass (**F**) all showed negative correlations with serum melatonin levels. Con: control group; DF: daytime feeding group; NF: nighttime feeding group; NL: non-lactating group. Data are presented as means ± standard error. Different letters indicate significant intergroup differences determined by Tukey’s post hoc test.

The serum melatonin levels in lactating striped hamsters were significantly lower than those in the NL group under the ad lib feeding conditions. Compared with the Con group, TRF led to an upward tendency in serum melatonin levels in the DF and NF groups, but no significant differences were observed among those lactating groups (*F*_(3,26)_ = 3.245, *p* < 0.05, Figure 6A). The level of serum melatonin showed negative correlations with food intake (*p* < 0.05, Figure 6B), average metabolic rate (*p* < 0.05, Figure 6C), nighttime metabolic rate (*p* < 0.05, Figure 6E), and liver mass (*p* < 0.05, Figure 6F), but not with daytime metabolic rate (*p* > 0.05, Figure 6D).

### 3.6. Mitochondrial Respiration

**Figure 7:**
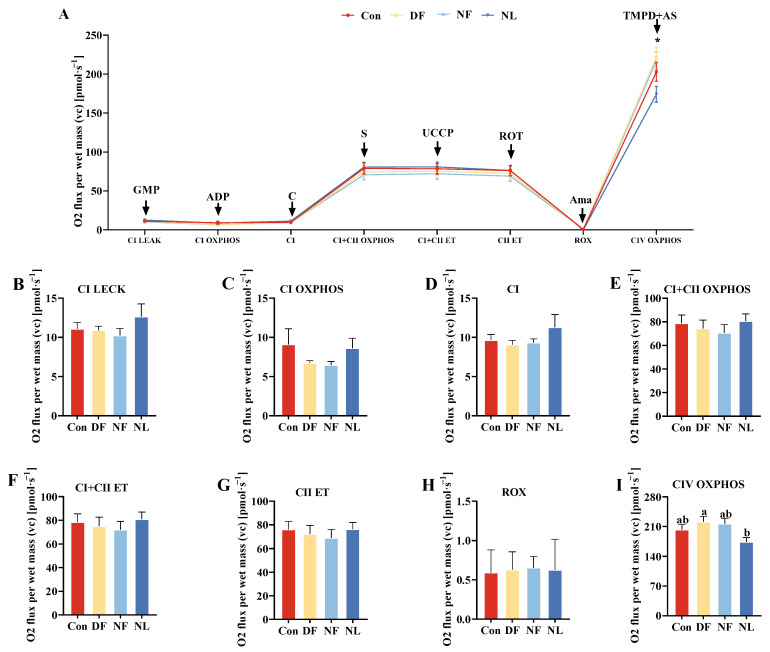
Effects of time-restricted feeding on mitochondrial respiration and reactive oxygen species production in female striped hamsters (**A**). Group averages are illustrated for tissue CI-linked substrate status (**B**), OXPHOS capacity of Complex I (**C**), Complex I status (**D**), OXPHOS capacity of Complex I and II (**E**), electron transfer system capacity of Complex I and II (**F**), electron transfer system capacity of Complex II (**G**), residual oxygen consumption (**H**), and OXPHOS capacity of Complex IV (**I**). DF females showed a higher level of OXPHOS capacity of Complex IV than NL females (**I**). con, control group; DF, daytime feeding group; NF, nighttime feeding group; NL, no lactation group. CI LEAK = complex I Leak respiration; CI OXPHOS = OXPHOS capacity of Complex I; CI + CII = OXPHOS capacity of Complex I and II; CI + II ET = electron transfer system capacity of Complex I and II; CII ET = the electron transfer system capacity of Complex II; ROX = residual oxygen consumption; CIV OXPHOS = OXPHOS capacity of Complex IV; PMG = pyruvate, malate, and glutamate; C = cytochrome c; S = succinate; Rot = rotenone; Ama = antimycin a; TMPD = trimethylpentanediol; AS = ascorbic acid. Data are presented as mean ± standard error. *, *p* < 0.05. Different letters indicate significant between-group differences determined by the Tukey post hoc test.

TRF had a certain effect on mitochondrial respiration and reactive oxygen species production in lactating striped hamsters (Figure 7A). The NF group had the higher OXPHOS capacity of Complex IV than the NL group (*F*_(3,23)_ = 3.353, *p* < 0.05, Figure 7I). There were no significant group differences in tissue CI-linked substrate status (*F*_(3,23)_ = 0.951, *p* > 0.05, Figure 7B), OXPHOS capacity of Complex I (*F*_(3,23)_ = 1.038, *p* > 0.05, Figure 7C), Complex I status (*F*_(3,23)_ = 1.072, *p* > 0.05, Figure 7D), OXPHOS capacity of Complex I and II (*F*_(3,23)_ = 0.41, *p* > 0.05, Figure 7E), electron transfer system capacity of Complex I and II (*F*_(3,23)_ = 0.305, *p* > 0.05, Figure 7F), electron transfer system capacity of Complex II (*F*_(3,23)_ = 0.261, *p* > 0.05, Figure 7G), and residual oxygen consumption (*F*_(3,23)_ = 0.038, *p* > 0.05, Figure 7H) among the groups.

### 3.7. Gut Microbiota

**Figure 8:**
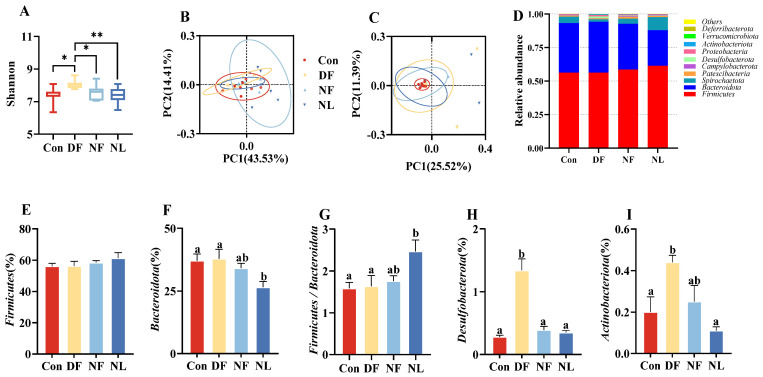
Effects of time-restricted feeding on the α diversity (**A**) and β diversity (**B**,**C**) of gut microbiota in female striped hamsters, as well as the abundance of the top 10 gut microbiota at the phylum level (**D**), the abundances of *Firmicutes* (**E**), *Bacteroidota* (**F**), *Firmicutes/Bacteroidete* ratio (**G**), *Desulfobacterota* (**H**), and *Actinobacteriota* (**I**). Con: control group; DF: daytime feeding group; NF: nighttime feeding group; NL: no lactating group. Data are presented as means ± standard error. * *p* < 0.05; ** *p* < 0.01. Different letters indicate significant intergroup differences determined by Tukey’s post hoc test.

The α-diversity of the gut microbiota in the DF group is significantly higher compared to the other three groups (*F*_(3,26)_ = 3.255, *p* < 0.05, Figure 8A). No significant differences in β-diversity were observed among the groups (Figure 8B,C). At the phylum level, the top 10 microbial taxa showed that there were no significant differences in the abundance of *Firmicutes* among the groups, while the abundance of *Bacteroidete* in lactating animals was lower than in non-lactating animals (*F*_(3,26)_ = 0.874, *p* > 0.05, Figure 8D,E; *F*_(3,26)_ = 3.833, *p* < 0.05, Figure 8D,F). In the NL group, the Firmicutes/Bacteroidota ratio of striped hamsters was significantly higher than that of the control group (*F*_(3,26)_ = 3.902, *p* < 0.05, Figure 8D,G). Additionally, the DF group showed significant increases in the abundance of *Desulfobacterota* (*F*_(3,26)_ = 27.836, *p* < 0.001, Figure 8D,H) and *Actinobacteriota* (*F*_(3,26)_ = 5.858, *p* < 0.01, Figure 8D,I).

### 3.8. Rhythmic Protein Expression in Liver

**Figure 9:**
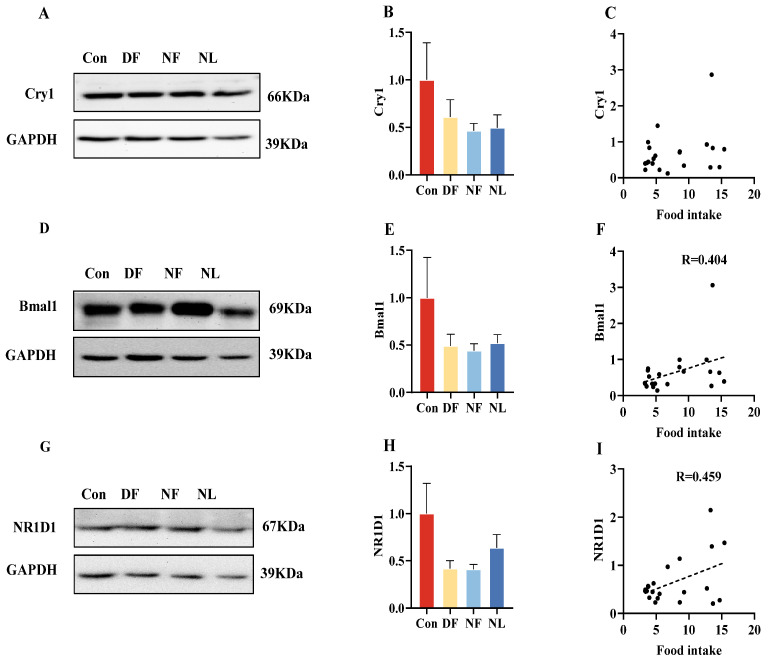
Effects of time-restricted feeding on Rhythmic protein expression in female striped hamsters. Western blot bands in the expression levels of Cry1 (**A**), with quantification of Cry1 expression (**B**), and correlation analysis of Cry1 and food intake (**C**). Western blot bands in the expression levels of Bmal1 (**D**) with quantification of Bmal1 expression (**E**) and correlation analysis of Bmal1 and food intake (**F**). Western blot bands in the expression levels of NRID1 (**G**) with quantification of NRID1 expression (**H**) and correlation analysis of NRID1 and food intake (**I**). Con: control group; DF: daytime feeding group; NF: nighttime feeding group; NL: non-lactating group. Data are presented as means ± standard error.

TRF resulted in a decreasing trend in Cry1 (*F*_(3,20)_ = 1.158, *p* > 0.05, Figure 9A,B), Bmal1 (*F*_(3,20)_ = 1.295, *p* > 0.05, Figure 9D,E), and NR1D1 (*F*_(3,20)_ = 2.316, *p* > 0.05, Figure 9G,H) protein expression in lactating striped hamsters in the DF and NF groups but did not reach statistical significance. In addition, there was a positive correlation between both Bmal1 (*p* < 0.05, Figure 9F) and NR1D1 (*p* < 0.05, Figure 9I) protein expression and food intake.

## 4. Discussion

Using the striped hamster model, we examined the effects of time-restricted feeding (TRF) on lactating females to test the hypothesis that TRF during lactation can have significant and phase-specific effects on bodily functions in females. Our data illustrate the adaptive changes and coping strategies of lactating hamsters under the TRF condition and highlight the importance of food access and dietary rhythm regulation in maternal and offspring health, development, and reproductive success.

### 4.1. Lactation Significantly Increased the Level of Energy Budget in Striped Hamsters

Lactation represents a critical period of energy requirement during which the mother must expend large amounts of energy and nutrients to maintain milk production and to display maternal care, placing a greater demand on the mother’s nutrient reserves and physiological status [31,46]. This notion is supported by our data showing that, compared to the NL females, Con females had increases in average food intake and oxygen consumption in striped hamsters, as reported in mice (*Mus musculus*) [47], rats (*Rattus norvegicus*) [48], hamsters (*Cricetulus barabensis*) [49], Brandt’s voles (*Lasiopodomys brandtii*, Radde, 1861) [50], large oriental voles (*Eothenomys miletus*, Thomas, 1914) [51], and Mongolian gerbils [52]. Our data also show that Con females had a larger mass of the small intestine than NL females. The small intestine is a primary organ for food digestion and absorption, and thus its increase represents a response to the increased food intake [53,54]. We also found that Con females had a higher level of body mass loss than the NL females. Body mass loss during lactation has been reported in C57BL/6J mice [55], Swiss mice [56], and Cotton rats (*Sigmodon hispidus*, Say and Ord, 1825) [57]. Lactating striped hamsters must coordinate energy intake and expenditure to meet the need for milk production and nursing [49], and, thus, the imbalance in energy regulation during lactation may lead to the continuous depletion of the mother’s energy reserves, causing a significant decline in body mass.

Interestingly, the Con females showed a lower level of circulating melatonin compared to the NL females, and circulating melatonin levels were significantly negatively correlated with food intake, average oxygen consumption, and liver weight, respectively. Several studies have demonstrated that melatonin can reduce food intake and energy expenditure. For example, exogenous melatonin administration has been reported to suppress food intake in male Wistar rats [58] and decrease oxygen consumption (an indicator of cellular energy expenditure) in mouse liver cells [59]. Melatonin can also regulate lipid and energy metabolism in the rat liver and alleviate hepatic hypertrophy [60,61]. Therefore, we propose that melatonin reduces liver weight by improving lipid metabolism and mitochondrial function [62]. Consistently, animals with higher melatonin levels tended to have relatively lighter livers in our study. In addition, melatonin is a critical hormone for circadian rhythms, and low levels of melatonin are often associated with sleep and rhythm disorders [63]. Therefore, we cannot exclude the possibility that the low melatonin in the Con females might be associated with their disrupted circadian rhythms related to frequent breastfeeding. Finally, the abundance of *Bacteroidetes* in the Con, compared to NL, hamsters also supports the finding that *Firmicutes* and *Bacteroidetes* are dominant phyla during reproductive stages in both humans and mice [64], although their functional roles during lactation are still unknown. The higher abundance of *Bacteroidetes* is also commonly associated with metabolic diseases [65].

### 4.2. TRF Manipulation Reduced the Energy Budget and Litter Survival of Lactating Hamsters

To balance the large demands for energy requirements, lactating females usually increase their food intake [46], which indeed is also the case for the striped hamsters reported in the previous [49] and present studies. Our data also indicate that under the TRF conditions, both DF and NF females showed a significant reduction in their average food intake, compared to the Con females. Detailed data comparisons (Figure 2F–H) also illustrate that both DF and NF females consumed the amount of food during their subjective feeding period comparable to the level under the Con condition. These data clearly indicate that 24-h/day food access is essential for the lactating striped hamster to eat a sufficient amount of food to meet their energy demands. It is interesting to note that DF and NF females also displayed trending, although not statistically significant, low levels of protein expression on some of the rhythmic genes in the liver, and positive correlations of Bmal1 and NR1D1 expression with food intake further support the notion that rhythm proteins play a crucial role in maintaining the body’s circadian rhythm and energy metabolism [66]. It has been shown that under light-dark conditions, Bmal1^−/−^ mice exhibited arhythmic feeding behavior, characterized by higher food intake during the light (inactive) period and lower intake during the dark period, resulting in lower body weight compared to wild-type mice [67,68]. Global deletion of NR1D1 in C57BL/6J mice resulted in the loss of their feeding rhythm in constant darkness [68,69]. The causal relationships among those proteins, food intake, and TRF need to be examined in subsequent studies with larger sample sizes.

Our data also illustrate significant impacts of TRF on reducing litter body mass and litter survival rate in the DF and NF groups, compared to the Con group. TRF or other kinds of reduction in food intake during lactation have been shown to have negative impacts on offspring growth and development [70,71,72]. It is note that in rats [73] and mice [74], they show a significant reduction in basal metabolic rate (BMR) when faced with food restriction. In contrast, TRF manipulation did not alter body mass, organ mass, metabolic rate, and complex IV function in lactating striped hamsters in the present study. We speculate that when lactating striped hamsters are exposed to environmental stressors, such as food limitation, they may prioritize protecting themselves by maintaining their own physiological functions for future reproduction when resource conditions improve [75].

### 4.3. Phase-Specific Effects of TRF Manipulation

In addition to the above-mentioned TRF effects, our data also show some phase-specific effects of the TRF manipulation. For example, compared to the Con group, DF lactating females showed increased body mass loss, a lower level of *CART* gene expression in the hypothalamus, and increased abundance in *Desulfobacterota* and *Actinobacteriota* in their gut microbiota. The striped hamsters are typical nocturnal animals that consume more than 2/3 of their daily food during nighttime. Therefore, the observed DF effects may primarily be attributed to food deprivation during the dark phase/day. The lack of food intake during their dark phase, together with dietary rhythm changes, in those lactating hamsters might have disrupted their rhythm to consume adequate energy and to produce sufficient milk for breastfeeding. In previous studies in rats and mice, alteration of maternal circadian rhythms was found to affect the development of mammary glands, ultimately leading to insufficient lactation [76,77]. When striped hamsters reduced food intake under 33 °C, their milk energy output was reduced to 78.1% associated with a 12.7% decrease in pup mass [78]. Therefore, insufficient milk production and nutrition with decreased maternal care could lead to the reduced litter size and pup survival observed in the present study. In addition, the phylum *Desulfobacterota*, a producer of immunostimulatory lipopolysaccharide (LPS), is positively correlated with IgG and may thus influence the intestinal microenvironment, gut barrier function, and host immune function [79,80]. Meanwhile, previous studies have reported that enriching the gut microbiome with *Actinobacteriota* (specifically, the *Bifidobacterium* genus) using prebiotics is positively correlated with improved glucose tolerance and insulin secretion [81]. In contrast, a reduction in *Bifidobacterium* abundance is likewise linked to various disease states [82]. Thus, under varying feeding conditions, shifts in these taxa may reflect differences in the hamsters’ health status and energy metabolic profiles. The increased *Desulfobacterota* and *Actinobacteriota* may further facilitate the observed DF effects—a speculation which needs to be tested in subsequent experiments.

While the DF females had a lower level of hypothalamic *CART* gene expression, the NF females showed lower levels of *AgRP* and *POMC* gene expression also in the hypothalamus. It has been amply demonstrated that these neuropeptides are involved in food intake and have bidirectional interactions with the behavior. For example, optogenetic activation of *AgRP* in the hypothalamus significantly increased food intake [83], whereas food shortage led to reduced digestive behavior and a reduction in the hypothalamic expression of *CART*, *AgRP*, and *POMC* in mice and Chaotung vole (*Eothenomys olitor*, Thomas, 1911) [84,85]. Our data indicate that the lack of food access and eating activity due to the TRF manipulation might have disrupted those neuropeptide gene expressions in the hypothalamus via the behavior-brain bidirectional interactions [83]. In addition, these neuropeptides may respond to altered dietary rhythms in a neuropeptide- and circadian rhythm-specific manner [86,87].

## 5. Conclusions

In summary, our data demonstrate that lactating striped hamsters can increase their energy budget by enhancing food intake, oxygen consumption, and small intestine mass, compared to the NL females. Consistent with our hypothesis, TRF disrupts the energy metabolism of lactating striped hamsters, as evidenced by significant decreases in food intake of lactating females and reductions in litter body mass and survival rate in the TRF group. TRF manipulation also showed some phase-specific (DF vs. NF) effects on lactating females by facilitating their body mass loss, reducing rhythmic gene expression in the hypothalamus, and increasing the abundance of *Desulfobacterota* and *Actinobacteriota* of gut microbiota. In lactation biology, lactation success depends not only on energy intake but also on the feeding rhythm, advancing our understanding of the maternal-offspring energy allocation strategies. Furthermore, feeding rhythm, a key environmental factor shaping lactation outcomes, may apply generally to species with temporal food constraints in nature. With regard to human health, our results underscore the importance of regular dietary patterns for lactating individuals, with specific attention to shift workers and other groups with irregular daily schedules. Together, these data illustrate adaptive changes and coping strategies of lactating hamsters under TRF conditions, and highlight the importance of food access and dietary rhythm regulation in maternal and offspring health, development, and reproductive success.

## Figures and Tables

**Table 1:** Gene-specific primer sequences used for real-time RT-QPCR analysis.

Gene	Primers (5′ to 3′)
*Actin* (forward)	AAAGACCTCTATGCCAACA
*Actin* (reverse)	ACATCTGCTGGAAGGTGG
*NPY* (forward)	ACCCTCGCTCTGTCCCTG
*NPY* (reverse)	AATCAGTGTCTCAGGGCTA
*AgRP* (forward)	TGTTCCCAGAGTTCCCAGGTC
*AgRP* (reverse)	ATTGAAGAAGCGGCAGTAGCAC
*CART* (forward)	TACCTTTGCTGGGTGCCG
*CART* (reverse)	AAGTTCCTCGGGGACAGT
*POMC* (forward)	GGTGGGCAAGAAGCGACG
*POMC*(reverse)	CTTGTCCTTGGGCGGGCT

NPY, neuropeptide Y; AgRP, agouti-related protein; CART, cocaine and amphetamine-regulated transcript; POMC, pro-opiomelanocortin.

## Data Availability

The original contributions presented in this study are included in the article. Further inquiries can be directed to the corresponding author.
